# A retrospective analysis of transudative pleural effusion due to fibrosing mediastinitis

**DOI:** 10.1186/s13019-024-02972-9

**Published:** 2024-07-26

**Authors:** Yan-Xia Yu, Li An, Zhan-Hong Ma, Huan-Zhong Shi, Yuan-Hua Yang

**Affiliations:** 1 Department of Respiratory and Critical Care Medicine, Beijing Institute of Respiratory Medicine and Beijing Chaoyang Hospital, Capital Medical University, 8 Gongti Nanlu, Chaoyang District, Beijing, 100020 China; 2 Department of Radiology, Beijing Chaoyang Hospital, Capital Medical University, Beijing, 100020 China

**Keywords:** Fibrosing mediastinitis, Transudative pleural effusion, Pulmonary vein stenosis, Diagnosis, Therapy

## Abstract

**Background:**

Pleural effusion caused by fibrosing mediastinitis is rarely reported. This study aimed to summarize the clinical manifestations, diagnosis and treatment of transudative pleural effusion due to fibrosing mediastinitis.

**Methods:**

Medical records and follow-up data of 7 patients with transudative pleural effusion due to fibrosing mediastinitis in Beijing Chaoyang Hospital between May 2014 and Feb 2018 were retrospectively analyzed.

**Results:**

These patients included 4 males and 3 females, with an average age of (64 ± 9) years. There were 3 left-sided effusions, 2 right-sided effusions and 2 bilateral effusions. Previous or latent tuberculosis was found in 6 patients. Pulmonary hypertension was indicated by echocardiography in all the 7 patients. Computed tomography pulmonary angiography (CTPA) of all the 7 cases showed increased soft tissue images visible in the mediastinum and bilateral hilus, different degrees of stenosis or occlusion in the pulmonary artery and pulmonary vein. In addition, 4 cases were found of right middle lobe atelectasis with a mediastinal window setting. There was interstitial pulmonary edema on the side of pleural effusion with a lung window setting. All the 7 patients were treated with intermittent drainage of pleural effusion combined with diuretic therapy. Five patients were treated with antituberculosis therapy. Up to now, two patients died of right heart failure and respiratory failure after 2 and 16 months respectively; The remaining 5 patients were still in follow up.

**Conclusion:**

Fibrosing mediastinitis can lead to pulmonary vein stenosis or occlusion, and thus cause transudative pleural effusion, which can be detected by CTPA. Pulmonary hypertension, long time of cough, and a history of tuberculosis are common in these patients. The common therapy is intermittent drainage of pleural effusion combined with diuretic therapy.

**Supplementary Information:**

The online version contains supplementary material available at 10.1186/s13019-024-02972-9.

## Introduction

Pleural effusion is a common clinical problem caused by different underlying diseases, such as cancer, congestive heart failure, hepatic hydrothorax, pulmonary embolism, nephrotic syndrome, etc. [1, 2]. The most common cause of malignant pleural effusion is lung cancer, followed by breast cance [1].

Fibrosing mediastinitis is often caused by the proliferation of acellular collagen and fibrous tissue within the mediastinum, and manifested with obstruction or compression of central airways, esophagus, or pulmonary veins or arteries [3]. The pulmonary vessels and tracheal bronchus stenosis or occlusion in patients of fibrosing mediastinitis often result in pulmonary hypertension and atelectasis [3, 4]. The focal type of fibrosing mediastinitis usually manifests as a localized, calcified mass in the paratracheal or subcarinal regions of the mediastinum or in the pulmonary hila under Computed tomography pulmonary angiography (CTPA) or magnetic resonance observations; The clinical images of the diffuse type show a diffusely infiltrating, which often noncalcified mass that affects multiple mediastinal compartments [3]. When acute mediastinitis occurs, high morbidity and mortality follow [4].

The transudative pleural effusion due to fibrosing mediastinitis is rarely reported. In a previous report, a 70-year-old man who was finally diagnosed of fibrosing mediastinitis combined with transudative pleural effusion was misdiagnosed of tuberculous pleurisy with his specified breathlessness and refractory left pleural effusion [5]. This report by Yang et al. [5]. indicated transudative pleural effusions of might be caused by the rare disease-fibrinous mediastinitis. Therefore, fibrosing mediastinitis combined with transudative pleural effusion requires careful diagnosis and etiological differentiation [5].

Therefore, we reviewed and analyzed the medical records and follow-up data of the 7 patients with transudative pleural effusion due to fibrosing mediastinitis in our hospital, in order to summarize the clinical manifestations, diagnosis and treatment for these rare cases.

## Methods

### Subjects

A retrospective analysis was made in 7 patients with transudative pleural effusion due to fibrosing mediastinitis in Beijing Chaoyang Hospital between May 2014 and Feb 2018. These patients had been admitted to hospital for transudative pleural effusion. The clinical manifestation, lab test, imaging analysis, pulmonary function, echocardiography, bronchoscopy, pulmonary artery angiography, and treatments were reviewed. The follow-up data were obtained by reviewing of the medical records and the telephone calls to patients and their families. The follow up ended in December of 2022.

This study was approved by the Medical Ethics Committee of our hospital. The written informed consents were obtained from the patients and the patients’ family members. The data in this study comply with the patient confidentiality principles.

### Diagnostic criteria for transudates

According to Light’s criteria [6]: (1) The ratio of pleural fluid protein to serum protein is greater than 0.5; (2) The ratio of pleural fluid Lactate dehydrogenase (LDH) and serum LDH is greater than 0.6; (3) Pleural fluid LDH is two-thirds greater than the normal upper limit for serum. Transudative pleural effusions meet none of these three characteristics. Etiology of the transudative pleural effusion had been confirmed of fibrosing mediastinitis, and excluded congestive heart failure, cirrhosis, nephrotic syndrome, hypoalbuminemia and other common diseases that can cause transudative pleural effusion.

### Diagnosis of fibrosing mediastinitis

The CTPA characteristics of fibrosing mediastinitis was either a diffuse or localized soft-tissue mediastinal mass. Typically, the soft-tissue mass obliterates normal mediastinal fat planes and causes compression of adjacent structures, including pulmonary vessels, trachea, esophagus, pericardium and heart, nerves and pleura, and even lung tissue [7]. The diagnosis of fibrosing mediastinitis was confirmed by CTPA in this study.

### Statistical analysis

SPSS Statistics version 22.0 (IBM, USA) was used in this study. Data were presented as mean ± standard deviation or number with percentage. Descriptive statistical methods were used for data analysis. Missing data was excluded for this study.

## Results

### Clinical manifestations

A total of 7 patients were studied, and the clinical manifestations of these patients were shown in Table 1. These patients included 4 males and 3 females, with an average age of (64 ± 9) years. The main symptoms were cough (7/7), breathlessness (7/7), blood in phlegm (1/7), and leg edema (2/7). The shortest time from onset of pleural effusion to making a definitive diagnosis was 40 days, and the longest was 8 months. The medium diagnosis time was 4.6 ± 2.6 months. There were 3 left-sided effusions, 2 right-sided effusions and 2 bilateral effusions. Six patients had a history of tuberculosis and one with a history of occupational dust exposure (iron and zinc).

**Table 1:** The medical history and general data of the 7 patients with transudative pleural effusion due to fibrosing mediastinitis

Patient number	Admission date	Gender	Age	Past medical history	Physical examinations	Diagnosed methods	Symptoms	Diagnosis duration (months)	Location of pleural effusion	Treatment	Follow up
1	May 2014	M	57	Tuberculosis	Loss of respiratory sounds in the right lower lung, coarse respiratory sounds in the left lung with dry and wet rales	CTPA	Cough and sputum for six years, short of breath, sputum with brown blood in it, bilateral lower extremity edema	Six	Right-sided	Sildenafil 25 mg qd, valsartan 80 mg qd, experimental quadruple anti-TB treatment, and multiple extractions of pleural fluid.	Died of right heart failure and respiratory failure 2 months later
2	Jun 2015	M	51	Tuberculosis, Bronchiectasis, Right lower lobe lung resection	Low breath sounds on the left side, clear breath sounds in the right lungs; No pathologic murmurs were heard. Heart rate 110 beats/min.	CTPA	Cough, short of breath for sixteen years; worsen with exertion, and sputum with blood in it	Five	Bilateral	Consider bronchiectasis and performed a right lower lobectomy	Died of heart failure and respiratory failure 16 months after discharge
3	May 2016	F	65	Tuberculosis, Hypertension	Decreased breath sounds in both lower lungs; edema of both lower extremities	CTPA	Cough, short of breath for 6 years; Weight loss of about 10 kg in 6 months; Hemoptysis and wheezing with low-grade fever	Three	Right-sided	Closed chest drainage was given for more than half a month, and about 500 ml of water was drained daily;	Alive
4	Sep 2016	F	66	Tuberculosis	Cardiac ultrasound suggests tricuspid regurgitation (mild), pulmonary hypertension (60 mmHg)	CTPA	Cough, short of breath, leg edema; Night sweats, loss of weight	One	Bilateral	Bilateral pleural effusions were drained separately	Alive
5	Feb 2017	M	62	Occupational dust exposure (iron and zinc)	Thick breath sounds in the right lung, decreased breath sounds in the left lower lung	CTPA	Cough, short of breath,	Seven	Left-sided	Anti-infective treatment, experimental anti-tuberculosis treatment, and thoracic injection of BCG polysaccharide ribonucleic acid, and the patient’s left pleural effusion was drained 800–1200 ml/d	Alive
6	Apr 2017	F	79	Tuberculosis, Diabetes, coronary atherosclerotic heart disease	Clear breath sounds on the right side and decreased breath sounds in the left lower lungs	CTPA	Cough, short of breath	Two	Left-sided	Thoracentesis was performed twice, and 900 ml of yellowish pleural fluid was withdrawn.	Alive
7	Feb 2018	M	70	Tuberculosis	Bilateral clear breath sounds, decreased breath sounds in the left lower lungs	CTPA	Cough, short of breath	Eight	Left-sided	Patient after drainage of pleural fluid (6000 ml), followed by recurrence of pleural fluid and multiple drains	Alive

M, Male; F, Female; TB, tuberculosis

In Supplemental Digital Content, the details about physical characteristics and pathologic indicators of each patient were displayed.

### Results of lab and assistant examinations

The results of lab and assistant examinations were shown in Table 2. In the 7 patients, the erythrocyte sedimentation rate was 12.7 ± 7.1 mm/h, purified protein derivative tests were all negative, and the T-SPOT.TB test was negative in 4 cases, and the other 3 cases were positive. N-terminal pro-brain natriuretic peptide was from 10 to 3957 pg/ml. Five patients had type I respiratory failure, and two had hypoxemia.

**Table 2:** The main laboratory and assistant examinations of the 7 patients with transudative pleural effusion due to fibrosing mediastinitis

Patient number	ESR mm/h	BNP pg/ml	PPD	T-spot (A:B)	Blood gas analysis	Pulmonary arterial systolic pressure by echocardiography (mmHg)	Pulmonary function	Bronchoscopy	Pulmonary angiography
1	8	3957	(−)	0; 0	Type I respiratory failure	126	Mixed ventilatory dysfunction	Multiple lobar and segment bronchus stenosis, RML occlusion, and scattered foci of black spots	Bilateral multiple branches of pulmonary artery stenosis
2	16	272.8	(−)	0; 0	Type I respiratory failure	96	Not performed	Not performed	Not performed
3	13	101.5	(−)	100; 0	Hypoxemia	50.7	Obstructive ventilatory dysfunction	Anterior segments of RUL, LUL bronchus stenosis, RML occlusion, scattered foci of black spots	Pulmonary artery of RUL and RML occlusion
4	7	750.3	(−)	60; 80	Type I respiratory failure	70	Not performed	Not performed	Not performed
5	10	363.1	(−)	0; 0	Hypoxemia	63	Obstructive ventilatory dysfunction	Multiple lobar and segment bronchus stenosis, RML occlusion, and scattered foci of black spots	Pulmonary artery of RUL and RLL stenosis
6	27	253.5	(−)	40; 200	Type I respiratory failure	50	Obstructive ventilatory dysfunction	Not performed	Not performed
7	8	10	(−)	0:0	Type I respiratory failure	63	Not performed	Not performed	Pulmonary artery of LUL and RLL stenosis

ESR, erythrocyte sedimentation rate; PPD, purified protein derivative; RUL, right upper lobe; RML, right middle lobe; RLL, right lower Lobe; LUL, left upper lobe; LLL, left lower lobe

Pulmonary hypertension was suggested by echocardiography in all the 7 patients, and pulmonary artery systolic pressure was 74.1 ± 27.6 mmHg. The patient with the highest pulmonary hypertension also had right ventricular enlargement, right ventricular hypertrophy, and decreased right ventricular wall motion. Pulmonary function showed obstructive ventilatory dysfunction in 3 cases and mixed ventilatory dysfunction in one case.

Bronchoscopy of 3 patients showed airway distortion, multiple stenosis or occlusion, and scattered foci of black spots in the airway mucosa. Two cases were examined by medical thoracoscopy, one case had no positive result, and the other case (case 5) showed scattered foci of black nodules in the visceral, parietal and diaphragmatic pleura. A diagnostic biopsy of the nodules suggested a granulomatous inflammation induced by small foreign bodies.

**Fig. 1:**
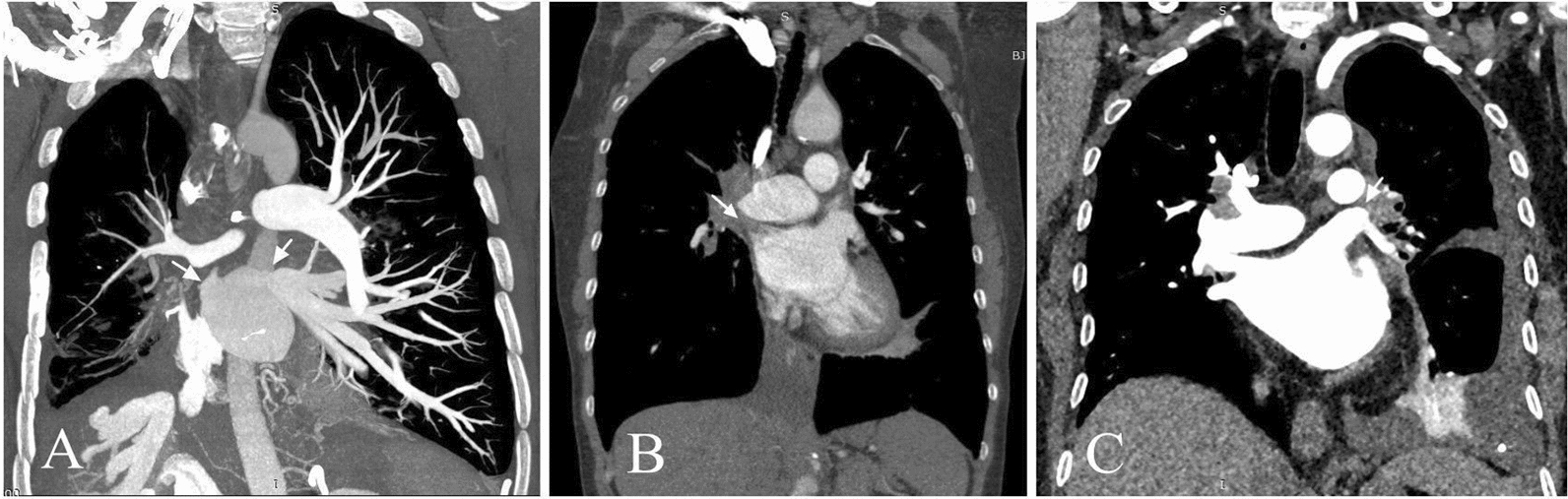
Coronal reformatted three-dimensional reconstructions images of computed tomography pulmonary angiography. Panel **A** shows right pulmonary vein and left superior pulmonary vein occlusion (arrow, case 2). Panel **B** shows right superior pulmonary vein occlusion (arrow, case 3). Panel **C** shows a main branch of left superior pulmonary vein occlusion (arrow, case 6)

**Fig. 2:**
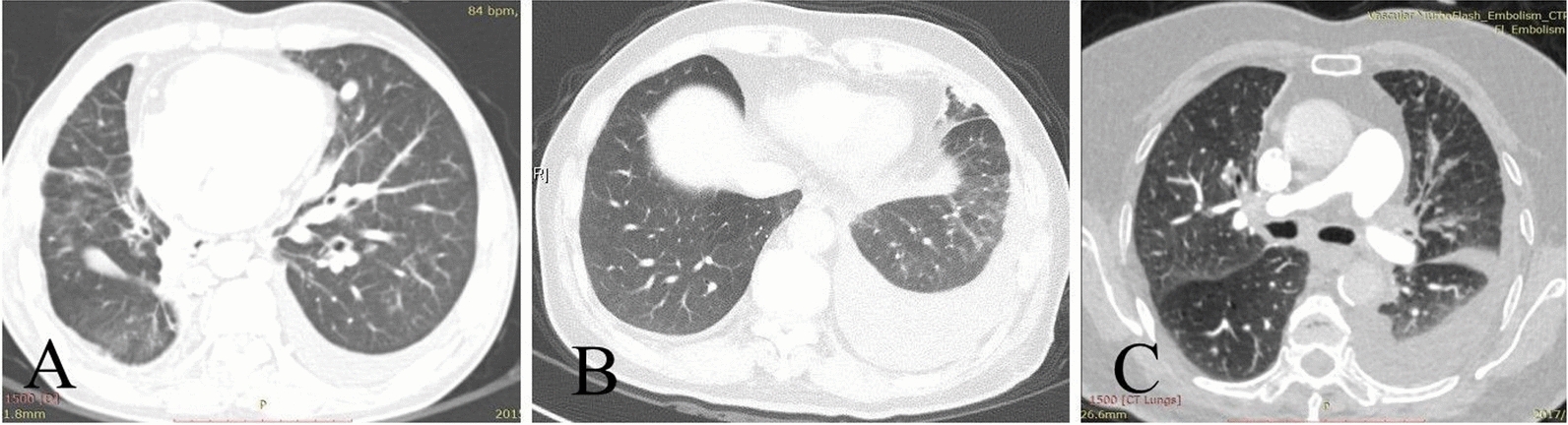
Computed tomographic scan of the chest. Panel **A** shows bilateral pleural effusions, scattered thickening of interlobular septa and ground-glass attenuation in the left lung (case 2). Panel **B** shows left-sided pleural effusion, scattered thickening of interlobular septa and patchy glass-like opacities in the left inferior lobe (case 5). Panel **C** shows left-sided pleural effusion, scattered thickening of interlobular septa in the left superior lobe (case 6)

**Fig. 3:**
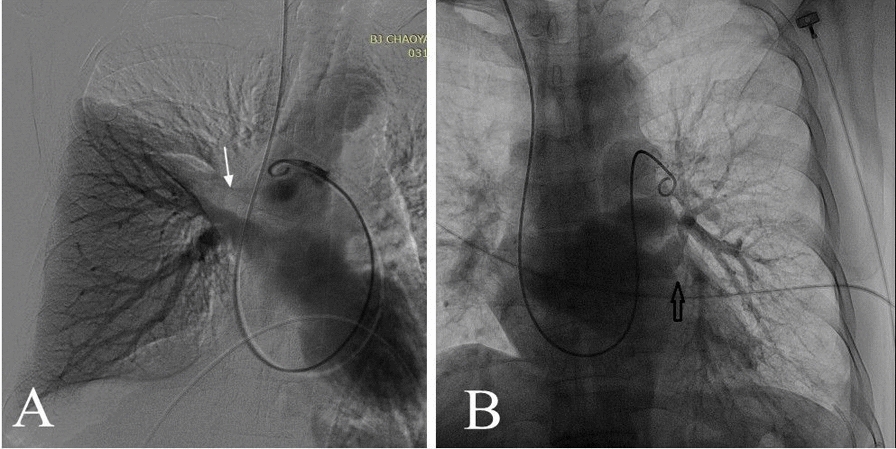
Findings on pulmonary artery angiography during vein period. Panel **A** shows right upper lung perfusion defect and right upper pulmonary vein without imaging (arrow, case 3). Panel **B** shows a main branch of the left inferior pulmonary vein is seen as a trumpet shaped development (arrow), indicating the pulmonary vein occlusion (case 5)

CTPA of 7 cases (Table 3) showed that increased soft tissue images were visible in the mediastinum and bilateral hilus, different degrees of stenosis or occlusion in the pulmonary artery and pulmonary vein (Fig. 1), and 4 cases with right middle lobe atelectasis with a mediastinal window setting; and there was interstitial pulmonary edema on the side of pleural effusion with a lung window setting (Fig. 2). Pulmonary arteriography was performed in 4 cases, indicating multiple pulmonary artery stenosis and/or occlusion during the artery period, and lung perfusion defect and pulmonary vein involvements without imaging during the vein period (Fig. 3).

**Table 3:** Manifestations of CTPA in the 7 patients with transudative pleural effusion due to fibrosing mediastinitis

Patient number	Mediastinal mass		Bronchus		Compression of pulmonary vessels		Interstitial pulmonary edema
	Diffuse/local	Calcification	Stenosis	Atelectasis	Artery	Vein	
1	Diffuse	−	+	+	+	+	+
2	Diffuse	−	+	−	+	+	+
3	Diffuse	−	+	+	+	+	+
4	Diffuse	+	+	−	+	+	+
5	Diffuse	+	+	+	+	+	+
6	Diffuse	+	+	−	+	+	+
7	Diffuse	+	+	−	+	+	+

+, Existent; −, nonexistent

### Treatment and follow-up

All 7 patients were treated with intermittent drainage of pleural effusion combined with diuretic therapy. Five patients were treated with antituberculosis therapy. Clinical follow-up was available in all the 7 patients, ranging from 2 to 47 months. Two patients died of right heart failure and respiratory failure at 2 (case 1) and 16 months (case 2) respectively after the diagnosis. The remaining 5 patients were treated with oral diuretics after discharge without repeating drainage of pleural fluid. They still had a small to moderate amount of pleural effusion that can be tolerated and received oral-take diuretics intermittently. They are still in follow-up.

## Discussion

The first question in assessing a patient with a pleural effusion is whether the effusion is a transudate or an exudate. The distinction between transudates and exudates can help to determine the underlying cause of a pleural effusion [1, 2]. Light’s criteria are the most used to separate transudates from exudates [6]. However, Light’s criteria on pleural effusions to classify transudative or exudative may have a misclassification rate of 10–20% [8, 9]. Pleural fluid met Light’s criteria for a transudate throughout in 6 of 7 patients. However, in the case 5 patient, LDH level (case 5) initially met exudate criteria, while in the later stage, the characteristics of pleural fluid were fully accordant with a transudate. We noted that only LDH met exudate at the early stage, whereas other parameters such as total protein, total nucleated cell and differentials counts were not typically consistent with exudate. While a large amount of pleural effusion (800–1200 mL per day) was drained daily for nearly 4 months, serum total protein and albumin were not reduced, indicating no obvious loss of protein. A protein gradient between the serum protein and pleural fluid was 3.24 g/dL at the early stage, so the pleural fluid initially should be a transudate.

The proliferation of fibrous tissue within the mediastinum is the obvious sign of fibrosing mediastinitis, which may lead to compression or obstruction of vital mediastinal structures [3, 4]. The most common cause for fibrosing mediastinitis is infection, which may be resulted from prior granulomatous infections [4]. Other causes include neoplasms, radiation therapy, immune-related diseases, traumatic hemorrhage, and drugs [3, 4]. An immune-mediated hypersensitivity response is presented to histoplasma capsulatum infection in most cases [4]. The pathogenesis of extensive fibrosing mediastinitis in Chinese patients is reported to be associated with tuberculosis (TB) infections [10]. In this study, 6 of 7 patients reported a history of tuberculosis. And in the TB test, 3 cases were still positive when they admitted to the hospital. However, it was difficult to get tissue from these sites including mediastinum and hilus pulmonic, all patients had no pathological diagnosis in this study, which is a limitation for these rare reports.

Most cases of fibrosing mediastinitis can be diagnosed based on clinical and radiographic criteria. A report summarized the chest radiographic imaging of fibrosing mediastinitis, that invasive, localized, and frequently calcified right-sided mediastinal masses were mostly revealed in the patients of this disease [11]. Typically, the soft-tissue mass obliterates normal mediastinal fat planes and causes compression of adjacent structures, including pulmonary vessels, trachea, esophagus, pericardium and heart, nerves and pleura, and even lung tissue, especially, pulmonary vein, because of the thin wall of the vein [7, 12]. In this report, the common features of the 7 patients with CTPA were that the soft tissue of the mediastinum and bilateral hilar markedly enlarged, accompanied by varying degrees of stenosis or occlusion in pulmonary artery and pulmonary venous, with or without atelectasis.

Pulmonary vein stenosis or occlusion due to fibrosing mediastinitis can result in an increase in hydrostatic pressure, which is the main cause of transudative pleural effusion. The imaging of pulmonary vein stenosis or occlusion can directly be seen on a CT angiogram of the chest [7, 12]. In addition, interlobular septal thickening and patchy ground glass opacities suggestive of interstitial pulmonary edema in lung window indirectly suggest pulmonary vein stenosis or occlusion. On the other hand, pulmonary angiography can help to diagnose pulmonary vein stenosis or occlusion. During vein period, involved pulmonary veins without imaging or thin can be seen. Meanwhile, the corresponding pulmonary field perfusion defect can also be found [7, 12]. In addition, pulmonary hypertension may severe as a common complication of the disease [13]. This study indicated pulmonary hypertension by echocardiography in all the 7 patients.

Treating the transudate due to fibrosing mediastinitis is particularly challenging. The underling diseases must be solved [1, 4]. Catheter-based intervention for fibrosing mediastinitis had an overall poor prognosis [14]. For those patients suffering from pulmonary vein stenosis or occlusion, catheter-based intervention, such as balloon angioplasty with or without a stent, is the potential treatment option, but the prognosis is poor. The Mayo Clinic reported eight patients with pulmonary vein stenosis or occlusion underwent balloon angioplasty or stents placed [14]. Four out of 8 patients died within four weeks of their first pulmonary vein intervention as reported in a previous study [14]. Therapy with antifungal, anti-inflammatory agents and antituberculosis provides little benefit; Glucocorticoids also do not appear to be beneficial [4, 10, 11, 15]. Therapy is recommended to be palliative, and the intermittent drainage of pleural effusion in combination with diuretic therapy is minimally invasive and inexpensive for the patient with thoracentesis drainage [16]. Previous report also showed effective drainage of pleural effusion in patients with pleural effusion followed by few complications [17]. In this study, all the 7 patients had not received pulmonary vein interventional treatment because of high risk and high cost. The causes of death of 2 patients during the follow-up were related to right heart failure. The other 5 patients were in stable condition and were still under follow-up.

This study was limited for its small sample size due to the rare occurrence of fibrosing mediastinitis. The incidence of pleural effusion due to pulmonary vein stenosis caused by fibrosing mediastinitis is very low, so that the sample size was small. More samples need to be collected to better understanding this disease in future.

In conclusion, fibrosing mediastinitis is a rare cause of transudative pleural effusion. For the patient with transudative pleural effusion who had a history of tuberculosis; or who had a long time of cough, phlegm with blood, and short of breath; or whose echocardiography suggests pulmonary hypertension; and whose radiographic imaging reveals the presence of interstitial pulmonary edema on the side of pleural effusion, with or without atelectasis, fibrosing mediastinitis should be considered. Fibrosing mediastinitis can be detected by CTPA. There is no guideline for therapy now.

### Supplementary Information

Supplementary Material 1.

## Data Availability

The datasets generated during and/or analyzed during the current study are available from the corresponding author on reasonable request.
